# Comparison Between Fractional Vegetation Cover Retrievals from Vegetation Indices and Spectral Mixture Analysis: Case Study of PROBA/CHRIS Data Over an Agricultural Area

**DOI:** 10.3390/s90200768

**Published:** 2009-02-02

**Authors:** Juan C. Jiménez-Muñoz, José A. Sobrino, Antonio Plaza, Luis Guanter, José Moreno, Pablo Martínez

**Affiliations:** 1 Global Change Unit, Imaging Processing Laboratory, University of Valencia, Paterna, 46980, Spain; E-mail: jcjm@uv.es, sobrino@uv.es; 2 Neural Network and Signal Processing Group, Computer Science Department, University of Extremadura, Cáceres, Spain; E-mail: aplaza@unex.es, pablomar@unex.es; 3 GeoForschungsZentrum Potsdam, Remote Sensing Section, Telegrafenberg, D-14473, Potsdam, Germany; E-mail: luisguan@gfz-potsdam.de; 4 Laboratory of Earth Observation, Imaging Processing Laboratory, University of Valencia, Paterna, 46980, Spain; E-mail: jose.moreno@uv.es

**Keywords:** Fractional Vegetation Cover, Vegetation Indices, Spectral Mixture Analysis, PROBA, CHRIS

## Abstract

In this paper we compare two different methodologies for Fractional Vegetation Cover (FVC) retrieval from Compact High Resolution Imaging Spectrometer (CHRIS) data onboard the European Space Agency (ESA) Project for On-Board Autonomy (PROBA) platform. The first methodology is based on empirical approaches using Vegetation Indices (VIs), in particular the Normalized Difference Vegetation Index (NDVI) and the Variable Atmospherically Resistant Index (VARI). The second methodology is based on the Spectral Mixture Analysis (SMA) technique, in which a Linear Spectral Unmixing model has been considered in order to retrieve the abundance of the different constituent materials within pixel elements, called Endmembers (EMs). These EMs were extracted from the image using three different methods: i) manual extraction using a land cover map, ii) Pixel Purity Index (PPI) and iii) Automated Morphological Endmember Extraction (AMEE). The different methodologies for FVC retrieval were applied to one PROBA/CHRIS image acquired over an agricultural area in Spain, and they were calibrated and tested against in situ measurements of FVC estimated with hemispherical photographs. The results obtained from VIs show that VARI correlates better with FVC than NDVI does, with standard errors of estimation of less than 8% in the case of VARI and less than 13% in the case of NDVI when calibrated using the *in situ* measurements. The results obtained from the SMA-LSU technique show Root Mean Square Errors (RMSE) below 12% when EMs are extracted from the AMEE method and around 9% when extracted from the PPI method. A RMSE value below 9% was obtained for manual extraction of EMs using a land cover use map.

## 1. Introduction

Knowledge of the biophysical characteristics of vegetation is necessary for describing energy and mass fluxes at the Earth's surface using Global Circulation Models (GCMs), water models, and carbon cycle models [32]. Basic data regarding the extent and dynamics of vegetation are still needed, and better assessment of natural or man-made changes in the vegetation cover of the Earth is crucial to understand the role of plant communities in climatic, hydrologic and geochemical cycles [17]. Fraction vegetation cover (FVC) is one of the main biophysical parameters involved in the surface processes, which is also a necessary requirement for Numerical Weather Prediction, regional and global climate modelling, and global change monitoring [1,29]. Remote sensing is an effective tool for observing the distribution and evolution of the FVC, which can be considered as an indicator of land degradation [26].

Although less attention has been paid, FVC can be also a key parameter in thermal remote sensing, since it is a basic parameter from which surface emissivities can be estimated. The knowledge of surface emissivities in thermal remote sensing is necessary in order to retrieve land surface temperatures with enough accuracy. An overview on how FVC can be used to retrieve surface emissivities can be found in Sobrino *et al.* [28].

Vegetation indices (VIs) and spectral mixture analysis (SMA) are the most frequently used techniques in remote sensing to estimate the FVC [5]. Other methods rely on the inversion of radiative transfer models, as presented among others in [14], or on Artificial Neuronal Networks [2]. In this paper we have focused on a comparison between VIs and SMA techniques, which have been applied to data acquired by the CHRIS (Compact High Resolution Imaging Spectrometer) instrument on board the ESA PRoject for On-Board Autonomy (PROBA) platform.

**Figure 1.**
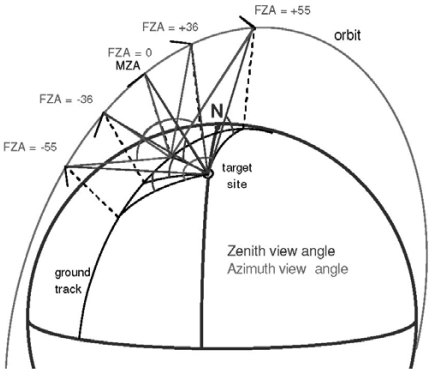
Schematic view of PROBA/CHRIS acquisition geometry.

The PROBA/CHRIS system, launched on the 22^th^ October 2001, is a technology demonstration experiment to take advantage of the autonomous pointing capabilities of a generic platform suitable for Earth Observation purposes. In combination, the coupled PROBA/CHRIS system [3] provides high spatial resolution hyperspectral/multiangular data, what constitutes a new generation of remote sensing information. On the one hand, the PROBA platform provides pointing in both across-track and along-track directions. In this way, the PROBA/CHRIS system has multiangular capabilities, acquiring up to five consecutive images from five different view zenith angles (VZA) in one single satellite overpass. Each imaged target has an associated “fly-by” position, which is the position on the ground track when the platform zenith angle, as seen from the target, is a minimum (i.e. Minimum Zenith Angle, MZA). The platform acquires the images at times when the zenith angle of the platform with respect to the fly-by position is equal to a set of Fly-by Zenith Angles (FZA): 0°, ±36° or ±55°. Negative MZA values correspond to target locations east of the ground track, while negative FZAs correspond to acquisition geometries when the satellite has already flown over the target position. A schematic view of PROBA/CHRIS acquisition geometry is displayed in Figure 1. On the other hand, CHRIS measures over the visible/near-infrared (VNIR) bands from 400 nm to 1,050 nm, with a minimum spectral sampling interval ranging between 1.25 (@400 nm) and 11 nm (@1,000 nm). It can operate in different modes, thus compromising the number of spectral bands and the spatial resolution because of storage reasons.

The paper is organized as follows: Section 2 describes the dataset employed throughout the paper. Section 3 provides a description of the methods for FVC retrieval that are based on empirical approaches with vegetation indices, as well as the testing against *in situ* measurements and a brief analysis of angular variations on vegetation indices and FVC depending on the CHRIS view angle. Section 4 describes the spectral mixture analysis and the linear spectral unmixing, with emphasis to the description of the methods considered to extract the endmembers, including also the testing against in situ measurements. Finally, Section 5 summarizes the results presented in the paper and concludes with some remarks and hints at plausible future research.

## 2. Dataset

### 2.1. PROBA/CHRIS data, test site and the SPARC field campaigns

The PROBA/CHRIS imagery used in the validation of the methodology comes from the dedicated ESA SPARC campaign [19]. It offered a unique situation in which PROBA/CHRIS images were acquired simultaneously with *in-situ* atmospheric and ground measurements.

**Figure 2.**
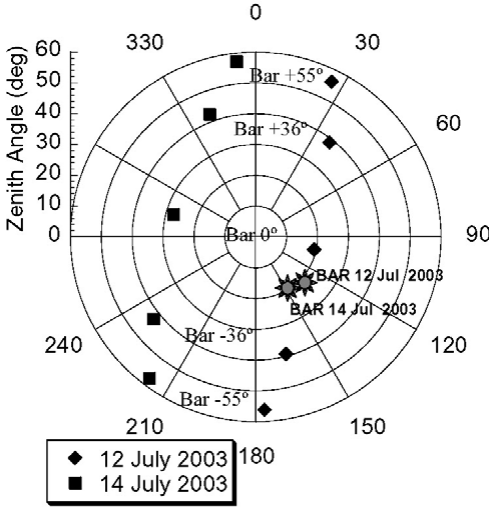
Acquisition geometries and illumination angles for the CHRIS/PROBA images acquired over Barrax on the 12th and the 14th of July 2003.

The first SPARC campaign took place in Barrax, La Mancha, Spain, from the 12th to 14th of July 2003, as part of Phase-A Preparations for the ESA SPECTRA mission. The reason for the selection of the 12-14th of July window was the coincidence with three consecutive days of PROBA/CHRIS overpasses. The situation over Barrax on those days was particularly favourable, because PROBA almost passed over (-4° across-track zenith angle) on July 13, and then again on July 12 (+20° across-track zenith angle) and on July 14 (-27° across-track zenith angle). Unfortunately, the image from July 13 was not correctly acquired because of satellite pointing problems, so we had only two images from the campaign. CHRIS images were acquired in Mode 1, with 62 spectral bands and 34 m/pixel as spatial resolution. The five acquisition angles for each of the two days are plotted in Figure 2. Previously to the atmospheric correction, the images were geometrically corrected before, drop-outs and striping noises were corrected as well.

**Figure 3.**
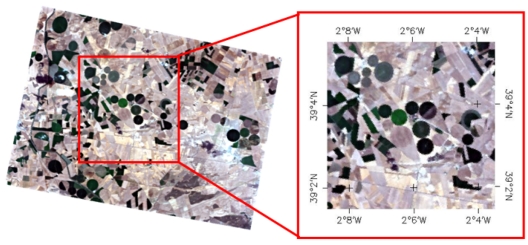
Test site as viewed by PROBA/CHRIS. The image shows a RGB composition in natural colour using CHRIS bands 25 (674.419 nm), 14 (563.373 nm) and 8 (501.531 nm). Green and dark tones are vegetated plots.

The Barrax site is a flat continental area with an average elevation over sea level of around 700 m. There is a big contrast in natural surfaces, ranging from green dense vegetation fields (e.g. potato crops) to dry, bare soils. The irrigation method in the region consists of circular pivots, which results in homogeneous large circular fields easily identifiable in the image. Besides, all the crops on the site have been classified previously, so a detailed map of the area with *in-situ* reflectance measurements, as well as several biophysical variables, is available. Figure 3 shows the Barrax test site as viewed by CHRIS.

### 2.2. Atmospheric correction

Concerning the atmospheric correction, the normal procedure in the processing of hyperspectral data consists in using atmospheric correction methods lying on a radiative transfer approach. Those usually start with the retrieval of the main atmospheric parameters from the data, using sophisticated algorithms to invert the measured Top-Of-Atmosphere (TOA) radiances. The accuracy of the retrievals is strongly conditioned by the spectral calibration of the instrument, and the subsequent surface reflectance as well.

However, since the PROBA/CHRIS system was designed as a technology demonstrator, radiometric performance is somehow limited for scientific applications. For this reason, PROBA/CHRIS 2003 and 2004 data (improvements for 2005 are foreseen) presents some mis-calibration trends all over the covered spectral region [8], with the most important one being the underestimation of the signal in the NIR wavelengths. As a result, common atmospheric correction methods would not lead to acceptable results. Within this framework, a dedicated atmospheric correction algorithm for PROBA/CHRIS data was designed [11]. Details can be found in the reference given. The basic idea is to combine both the radiative transfer and the empirical line approaches to atmospheric correction, in order to derive the appropriate atmospheric parameters and a set of correction factors for CHRIS's gain coefficients altogether. One of the strongest points of the method is that it works in a fully automatated manner, without the need for any ground-based atmospheric or surface reflectance ancillary information.

### 2.3. In-situ measurements

**Figure 4.**
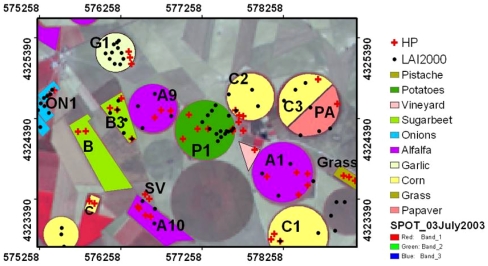
Land use map for the Barrax test site. Red crosses indicate the points where hemispherical photographs (HP) were taken.

**Figure 5.**
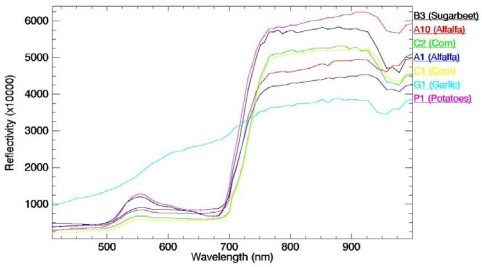
At-surface reflectivity spectra extracted from CHRIS image for the different samples (see Table 1).

FVC was estimated from ground measurements using a hemispherical digital camera. One of the main interest of hemispherical photographs is that the camera can be used under the canopy for upward and downward looking. Futhermore, the use of fish-eye lens allows the gap fraction to be evaluated in all viewing directions, which increases the accuracy of the derived FVC. Once properly classified, hemispherical photographs provide a detailed map of sky/soil visibility and obstruction. In turn, solar radiation regimes and canopy characteristics can be inferred from this map of sky geometry. The sampling strategy to be followed was designed according to statistical requirements. The dimension of the ESUs (Elementary Sample Units) selected was approximately 20 × 20 m^2^, and according to statistical requirements, between 4 and 15 ESUs were necessary to fully characterize the crop. Detailed information about the spatial sampling strategy, the measuring method and the hemispherical photograph processing can be found in [18]. Table 1 shows the mean values and the standard deviation for the FVC measured over the different crops by using hemispherical photographs, whereas Figure 4 shows the land use map for the Barrax test site with the ESUs marked. Figure 5 includes the mean at-surface reflectivity spectra extracted from the CHRIS image for the whole plots associated to the different crops considered in this study (see Figure 4 and Table 1).

## 3. Derivation of FVC from Vegetation Indices

### 3.1. Normalized Difference Vegetation Index (NDVI)

FVC has been traditionally estimated from remote sensing data using empirical relations with vegetation indices, as for example the Normalized Difference Vegetation Index (NDVI) [24], given by

(1) $$\text{NDVI}=\frac{\rho_{\text{nir}}-\rho_{\text{red}}}{\rho_{\text{nir}}+\rho_{\text{red}}}$$

where ρ_nir_ and ρ_red_ are the at-surface reflectivities obtained from sensor bands located in the near infrared (NIR) and red spectral regions. PROBA/CHRIS bands 48 (0.852 μm, NIR) and 25 (0.674 μm, red) can be then used in to obtain the NDVI. Table 2 shows the mean NDVI values extracted from the CHRIS image for the crops characterized with in situ measurements (see Table 1).

It has been demonstrated that FVC follows a linear relationship with the NDVI, for example using the concept of scaled NDVI [12]:

(2) $$\text{FVC}=\frac{\text{NDVI}-{\text{NDVI}}_{s}}{{\text{NDVI}}_{v}-{\text{NDVI}}_{s}}$$

where NDVIs and NDVIv correspond to representative values of NDVI for bare soil (FVC=0) and a vegetation (FVC=1), respectively. Other relationships, such as quadratic expressions have been also proposed [6], but they do not improve the results as discussed by Wittich and Hansing [33]. Note that Eq. (2) could be also expressed simply as typical linear relationship according to:

(3) FVC=a×NDVI+b

with *a* the slope and *b* the intercept given by:

(4) $$\begin{array}{r} a=\frac{1}{{\mathrm{NDVI}}_{v}-{\mathrm{NDVI}}_{s}}\\ b=\frac{{\mathrm{NDVI}}_{s}}{{\mathrm{NDVI}}_{v}-{\mathrm{NDVI}}_{s}} \end{array}$$

The main problem when applying Eq. (2) is the correct identification of NDVIs and NDVIv values. This is a critical task, so these values are region- and season-specific. Hence, for global studies with very low spatial resolution data (0.15°×0.15°), Gutman and Ignatov [12] proposed NDVIs = 0.04 ± 0.03 and NDVIv = 0.52 ± 0.03, which correspond to minimum and maximum values of the desert and evergreen clusters, respectively. Sobrino and Raissouni [27] considered a similar value for NDVIv (0.5), but a NDVs value of 0.2. We have analysed mean values of soil NDVI using the measured spectra of samples included in the ASTER spectral library (http://speclib.jpl.nasa.gov). For example, when using 44 soil samples belonging to different classes (alfisol, aridisol, entisol, inceptisol and mollisol) a value of NDVIs = 0.13 ± 0.09 was obtained, whereas when only seven soil samples belonging to the inceptisol class (the most abundant on Earth) were used a value of NDVIs = 0.19 ± 0.04 was obtained. These estimations and the different published values indicate that the NDVIs value ranges between 0 and 0.2, and most probably between 0.1 and 0.2 according to the results obtained from the ASTER library. This suggest that a mean value of NDVs=0.15 could be appropriate in most cases.

Regarding the NDVIv value selection, the value of 0.5 could be appropriate only when working with very low resolution data (typically > 10 km), but this value seems to be too low when using other higher resolution data (typically < 1 km). Using the vegetation samples included in the ASTER spectral library (excluding the dry grass sample), a value of NDVIv = 0.801 ± 0.012 has been obtained. This value is similar to the maximum NDVI values presented in Table 2 for the different crops. Since any of the crops included in Table 2 has exactly a FVC of 100%, an even higher NDVIv value is expected. For example, a maximum value of 0.91 was obtained for the whole CHRIS image. Therefore, when there is not *a priori* knowledge of the NDVIs and NDVIv values, mean values of 0.15 and 0.9 could be respectively considered as a reasonable basis.

**Figure 6.**
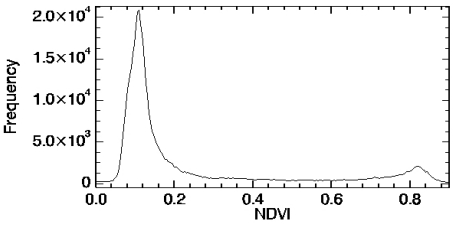
NDVI histogram extracted from the CHRIS image.

Some approaches have been also proposed to retrieve NDVIs and NDVIv values from image statistics. One of these approaches consists of choosing the minimum and maximum NDVI values for the whole scene as NDVIs and NDVIv. This approach assumes that pixels with FVC=0 and pixels with FVC=1 exists throughout the image. Despite that the assumption NDVIv=NDVImax could be appropriate, a special care should be taken when assuming NDVIs=NDVImin, since in most cases this values could be negative due to the presence of water bodies or other surfaces with negative NDVI values. In fact, a value of NDVImin=-0.14 was obtained for the CHRIS image considered in this study (as stated above, a NDVImax=0.91 was obtained). The other approach consists of retrieving the NDVIs and NDVIv values from the NDVI histogram. When enough bare soil and full-vegetated pixels exist on the image, the NDVI histogram shows two characteristic peaks which can be associated to the NDVIs and NDVIv values. Figure 6 shows the NDVI histogram extracted from the CHRIS image. The first peak at low NDVI values can be identified as NDVIs, with a value of 0.11 in this case, whereas the second peak at high NDVI values can be identified as NDVIv, with a value of 0.82 in this case. Note that the first peak (NDVIs) is clearly observed, whereas the second peak (NDVIv) is more smooth and less pronounced. This suggests that probably the first peak is a reasonable estimation of the NDVIs value, but NDVIv could be assumed to be the maximum NDVI value in the image, as a kind of combination of the two approaches discussed in this paragraph. A sensitivity analysis regarding the errors on FVC due to uncertainties on NDVIv and NDVIs can be found in Jiménez-Muñoz *et al.* [13]. The different approaches to retrieve the FVC from the NDVI will be tested and calibrated against *in situ* measurements in Section 3.3.

### 3.2. Green Vegetation Index

Despite that the NDVI has been widely used for assessment and monitoring of changes in canopy biophysical properties such as FVC, this vegetation index shows saturation problems for high vegetation covers, as has been pointed out by Gitelson *et al.* [10]. The authors found that for FVC higher than 60% the NDVI is almost insensitive to FVC changes, mainly due to the NIR reflectance behaviour. In order to solve this problem, NIR reflectances are substituted by green reflectances, thus developing a Green Vegetation Index (GVI) according to [10]:

(5) $$\text{GVI}=\frac{\rho_{\text{green}}-\rho_{\text{red}}}{\rho_{\text{green}}+\rho_{\text{red}}}$$

At-surface reflectivities obtained from PROBA/CHRIS bands 14 (0.563 μm, green) and 25 (0.674 μm, red) can be then used in to obtain the GVI. To reduce the atmospheric effects, the GVI given by Eq. (5) was modified using the concept of ARVI (Atmospherically Resistant Vegetation Index) [16]. Hence, Gitelson *et al.* [10] proposed a Variable Atmospherically Resistant Index (VARIgreen) given by:

(6) $${\text{VARI}}_{\text{green}}=\frac{\rho_{\text{green}}-\rho_{\text{red}}}{\rho_{\text{green}}+\rho_{\text{red}}-\rho_{\text{blue}}}$$

where ρ_blue_ refers to the reflectivity in the blue region, which can be obtained in this case from CHRIS band 8 (0.502 μm). GVI and VARIgreen are equivalent, but VARIgreen was designed only to introduce an atmospheric self-correction, so it is important to note that GVI is computed from at-surface reflectivities whereas VARIgreen is computed from TOA reflectivities. We have also considered the possibility of using a VI computed in the same way as VARIgreen but using at-surface reflectivities. This VI will be referred as Green Blue Vegetation Index (GBVI). Therefore, its expression is the same as Eq. (6) but using at-surface reflectivities. Table 3 shows the mean GVI, VARIgreen and GBVI values extracted from the CHRIS image for the crops characterized with in situ measurements (see Table 1).

Gitelson *et al.* [10] proposed a linear relationship between FVC and VARIgreen according to:

(7) $$\text{FVC}=a\times {\text{VARI}}_{\text{green}}+b$$

with a standard error of estimation less than 10%. Coefficients *a* and *b* are site specific, so they need to be recalculated for different study areas. We will show our particularized results in Section 3.3.

**Figure 7.**
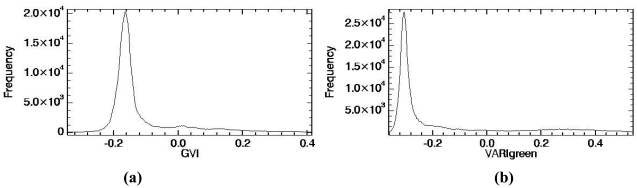
**(a)** GVI and **(b)** VARIgreen histograms extracted from the CHRIS image.

Following the procedure described in the previous section for the NDVI, we have also considered a scaled GVI, a scaled VARIgreen and a scaled GBVI to retrieve the FVC, i. e.:

(8) $$\text{FVC}=\frac{\text{GVI}-{\text{GVI}}_{s}}{{\text{GVI}}_{v}-{\text{GVI}}_{s}}$$

(9) $$\text{FVC}=\frac{{\text{VARI}}_{\text{green}}-{\text{VARI}}_{\text{green},s}}{{\text{VARI}}_{\text{green},v}-{\text{VARI}}_{\text{green},s}}$$

(10) $$\text{FVC}=\frac{\text{GBVI}-{\text{GBVI}}_{s}}{{\text{GBVI}}_{v}-{\text{GBVI}}_{s}}$$

where the subindices “v” and “s” refer to representative values for vegetation and soil. Equations (8), (9) and (10) could be also expressed as a linear relationship like the one given by Eq. (7), where slope “a” and intercept “b” are given by the same expression as Eq. (4) but substituting NDVI by GVI, VARIgreen or GBVI. As mentioned, a scaled GVI, VARIgreen or GBVI for FVC retrieval is proposed for the first time in this paper, so there are no published values for GVIv and GVIs (or VARIgreen,v and VARIgreen,s, or GBVIv and GBVIs). When computing the histogram for these indices, we have not found two characteristic peaks, as was the case of the NDVI. This result is presented in Figure 7 for GVI and VARIgreen, in which only one peak is observed. This peak is centred at -0.16 for the GVI and -0.31 for the VARIgreen. Minimum and maximum values throughout the CHRIS image were respectively -0.34 and 0.41 for GVI, and -0.36 and 0.54 for VARIgreen. Only one peak was observed also for the GBVI (not represented in Figure 7), centred at -0.24, and whith minimum and maximum values of -0.45 and 0.49, respectively. In next section (algorithms testing) we analyze if the minimum and maximum values could be chosen as representative values for soil and vegetation.

### 3.3. Algorithms testing

**Figure 8.**
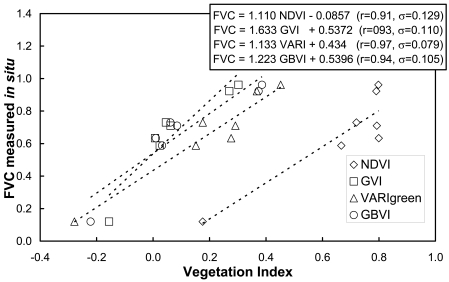
Empirical approaches between different vegetation indices and the fractional vegetation cover measured *in situ*. Fitted lines, correlation coefficients (r) and standard errors of estimation (σ) are also represented.

FVC retrievals using the different VIs discussed in the previous sections have been compared against the *in situ* measurements (Table 1) to assess the accuracy of the different approaches. Firstly, the VIs have been calibrated against the *in situ* FVC measurements to assess which VI provides the best correlation coefficient (r) and the minimum standard error of estimation (σ). The results obtained are represented in Figure 8, in which linear relationships like the ones given by Eq. (3) for the NDVI or Eq. (7) for the VARIgreen (and same for GVI or GBVI) have been considered. The best results (highest r and lowest σ) have been obtained for the VARIgreen, with σ < 8%, in accordance with the results presented by Gitelson *et al.* [10]. Over our study area, values of a=1.133 and b=0.434 for Eq. (7) have been obtained, significantly different from those obtained by Gitelson *et al.*, since as was commented in the previous Section, these coefficients are sensor and site specific. Note that the worst results were obtained for the NDVI approach, despite the fact that the error is still moderate, σ < 13%. Similar results were obtained with the GVI and GBVI, with σ = 11%. Note also that surprisingly the inclusion of the blue band and the use of TOA reflectivities (as is the case of the VARIgreen) improves the FVC retrievals in comparison with the GVI, which not uses the blue band since it is computed from at-surface reflectivities. This result was also found by Gitelson *et al.*, and there is not a satisfactory explanation for this fact.

Linear fits presented in Figure 8 have not been tested against an independent set of *in situ* measurements, since only seven samples (Table 1) were available and all of them were used to obtain the relations between FVC and VIs. Instead, FVC has been retrieved from the CHRIS image using the scaled NDVI, GVI, VARIgreen and GBVI given respectively by Equations (2), (8), (9) and (10) and compared to the *in situ* measurements. To this end, different combinations of ‘soil’ and ‘vegetation’ values associated with each VI have been considered. In the case of NDVI, ‘soil’ and ‘vegetation’ values have been extracted from the histogram, from minimum and maximum values, from a combination of histogram and maximum values and also assuming a standard or ‘global’ values according to the discussion presented in Section 3.1. In the case of GVI, VARIgreen and GBVI, we have only considered minimum and maximum values and a combination between histogram and maximum values, since it is not possible to obtain “vegetation” values from the histogram, and no global values have been published in the literature. In all the cases (NDVI, GVI, VARIgreen and GBVI) we have also included a selection of ‘soil’ and ‘vegetation’ values based on *in situ* measurements. These in situ values have been obtained using Eq. (4) (in the case of NDVI, and the analogous expression for the rest of VIs) and slope (a) and intercept (b) values presented in Figure 8. The results obtained are summarized in Table 4, in which bias (retrieved value minus *in situ* one), standard deviation of the bias (stdev), and Root Mean Square Error (RMSE, obtained as square sum of bias and stdev) are provided. Note that when *in situ* values are considered, a zero bias is obtained, and then stdev and RMSE are equal to the standard error of estimation (σ) presented in Figure 8. Although this is a kind of redundant information, we have also included in Table 4 these results to compare if the ‘soil’ and ‘vegetation’ values extracted from image data agree with the ones obtained from the *in situ* measurements. Apart from the results obtained from the *in situ* measurements, and in the same way as occurred when calibrating VIs against the in situ measurements (Figure 8), the best results are obtained in the case of the VARIgreen, with RMSE = 8% when VARIgreen,s and VARIgreen,v are associated with the minimum and maximum values of the image, respectively. When VARIgreen,s is extracted from the histogram, the RMSE raises to 10% due to an increase of the bias. In the case of GVI and GBVI, better results are also obtained when extracting ‘soil’ and ‘vegetation’ values from minimum and maximum image values, instead of choosing the ‘soil’ values from the histogram. Hence, in the case of GVI the RMSE increases from 16% to 27% when using the histogram. The GBVI provides slightly better results than GVI, with an increase on the RMSE from 13% to 22%. On the contrary, the NDVI approach provides better results when extracting ‘soil’ values from the histogram and ‘vegetation’ values from maximum image values than when extracting these values directly from minimum and maximum image values. Hence, if NDVIs is extracted from the histogram but NDVIv is chosen as the maximum image value, the RMSE is 13%. The same result is obtained considering global values of NDVIs=0.15 and NDVIv=0.90 (discussed in Section 3.1), and also NDVIs and NDVIv values obtained from the *in situ* measurements. When NDVIs and NDVIv are chosen as the minimum and maximum image values, the RMSE increases to 17%. The worst result for the case of the NDVI is obtained when both NDVIs and NDVIv are extracted from the histogram, with RMSE = 19%. Note that the NDVI approach tends to overestimate the FVC (positive bias), whereas the indices constructed with the green band (GVI, VARIgreen and GBVI) tend to underestimate (negative bias) the FVC. This result suggests that a fusion of VIs could be considered to improve the estimations, as proposed by Kallel *et al.* [15].

We would like to add that FVC was retrieved also using a NDVI computed from TOA reflectivities, and then compared to FVC retrieved with the NDVI computed from at-surface reflectivities, in order to assess the sensitivity to the atmospheric correction. As an example, in our test image and using the histogram to extract NDVIs and NDVIv, this difference (FVC from TOA NDVI minus FVC from at-surface NDVI) provided a mean value (bias) of -0.01, with a standard deviation of 0.02, therefore leading to a RMSE = 2.2%. Since FVC is not directly retrieved from the NDVI values but from a scaled NDVI, the final FVC retrieval seems to be not quite affected by the atmospheric effect. However, global values NDVIs=0.15 and NDVIv=0.90 refers to NDVI calculated from at-surface reflectivities. It would not be possible to establish global values in the case of a NDVI computed from TOA reflectivities, since NDVIs and NDVIv will depend on the atmospheric conditions.

**Figure 9.**
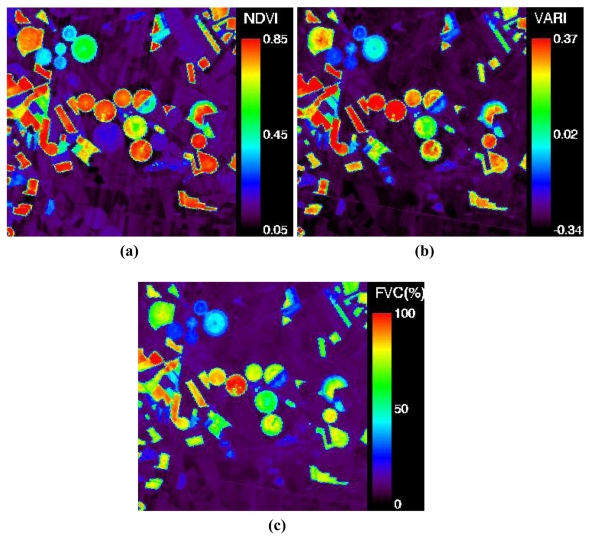
**(a)** Normalized Difference Vegetation Index (NDVI), **(b)** Variable Atmospherically Resistant Index (VARI) and **(c)** Fractional Vegetation Cover (FVC) retrieved from VARIgreen. Maps obtained from PROBA/CHRIS image acquired at near nadir view (see Figure 3).

As an example, Figure 9 shows the CHRIS image of NDVI and VARIgreen, and the final FVC retrieved from the VARIgreen approach using the in situ based values of VARIgreen,s and VARIgreen,v.

### 3.4. Angular sensitivity

**Figure 10.**
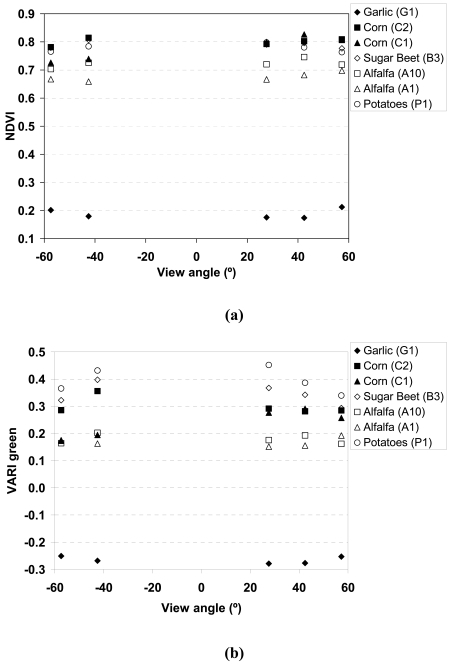
**(a)** NDVI and **(b)** VARIgreen versus the CHRIS view angle for different samples.

**Figure 11.**
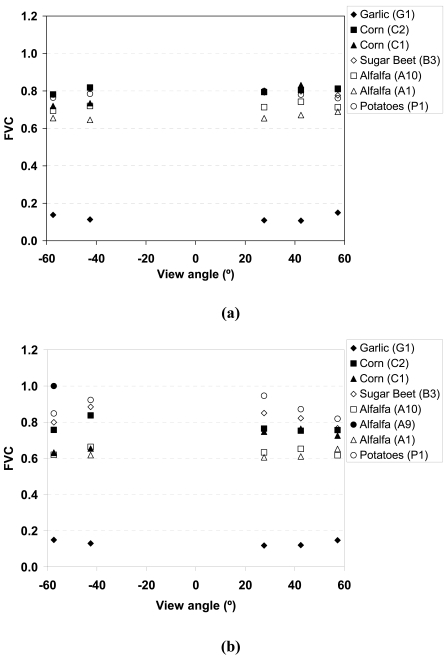
Fractional Vegetation Cover (FVC) retrieved from **(a)** NDVI and **(b)** VARIgreen versus the CHRIS view angle for different samples.

Despite that it is not the main objective of this paper, we have also roughly analysed the angular sensitivity of the VIs, focusing only on NDVI and VARIgreen, and its impact on the FVC retrieval. For this purpose, values have been extracted for each plot at the five PROBA/CHRIS acquisition view zenith angles: -57.40°, -42.53°, 27.60°, 42.44° and 57.29°. Figure 10 shows the angular variation of the NDVI and the VARI_green_ extracted from the seven plots considered in this study (see Table 1), and Figure 11 shows the angular variation on the FVC retrieved from these two VIs (using the expressions obtained from *in situ* measurements, presented in Figure 8). Percentage of FVC variations from the nadir value are provided in Table 5. The highest angular variations on FVC are obtained for the garlic (G1) crop, since it has the lowest FVC values and then the increase on the FVC with an increasing view angle is more pronounced. However, for the rest of crops, mainly for alfalfa and corn (C1, C2, A1, A10) with FVC measured values ranging from 59 to 73%, a strange behaviour is observed, since in some cases a lower FVC leads to a higher angular variation but in other cases this is not observed. This fact could be explained due to the different angular response of VIs at backward and forward directions, as is pointed out in the case of NDVI by Vercher *et al.* [30]. For the crops with the highest FVC (sugarbeet, B3, and potatoes, P1), with FVC > 90%, a decrease on the FVC with an increasing view angle is observed. When comparing FVC from NDVI and FVC from VARIgreen, a higher angular sensitivity was found in the case of the VARIgreen. Low variations on NDVI with the view angle were also obtained by Galvao *et al.* [9]. These authors pointed out that the higher angular variations on NDVI are due to changes in the solar zenith angle, and not in the view angle.

Further research dealing with the angular sensitivity of these VIs (overall for the VARIgreen) and the FVC retrieved from them is required to extract stronger conclusions. A more detailed sensitivity analysis of vegetation indices derived from CHRIS data can be found in Verrelst *et al.* [31], although that work only focuses on VIs and not on FVC retrievals, and VIs constructed with a green band are not considered either.

## 4. Derivation of FVC from Spectral Mixture Analysis: case of Linear Spectral Unmixing

The Spectral Mixture Analysis (SMA) technique has been developed in recent years to extract land-cover information at a sub-pixel level. SMA divides each ground resolution element into its constituent materials using endmembers (EMs), which represent the spectral characteristics of the cover types. When applied to multispectral satellite data, the result is a series of images each depicting the abundance of a cover type. The basic physical assumption is that there is not a significant amount of photon multiple scattering between the macroscopic materials, in such a way that the flux received by the sensor represents a summation of the fluxes from the cover types (macroscopic materials) and the fraction of each one is proportional to its covered area [5]. This assumption complies with the properties of the considered CHRIS/PROBA data sets, collected over a flat area and dominated by homogeneous crop fields. As a result, most of the endmember substances are sitting side-by-side within the field of view of the imager, and minimal secondary reflections or multiple scattering effects can be assumed. In this paper a simple linear mixing model LSU (Linear Spectral Unmixing) has been used, in which only a few EMs are used to describe the surface composition in each pixel of an image. Each EM is the spectral representation of a basic constituent in the scene. The general form of the LSU models is [25]:

(11) $$\rho_{i}=\sum_{em=1}^{Ne}F_{em}\rho_{em,i}+E_{i};\;\sum_{em=1}^{Ne}F_{em}=1$$

where ρ_i_ is the reflectivity for each channel (i), Ne is the number of EM (less or equal to the number of image channels), F_em_ is the fraction of EM and E_i_ is the unmodeled residual. The E_i_ term is commonly combined as the root mean square (rms) residual over all image channels (M):

(12) $$\text{rms}={\left(M^{-1}\sum_{i=1}^{M}E_{i}^{2}\right)}^{0.5}$$

In this study the reflectivity spectra for each endmember have been extracted from the image using different methods, which included EM extraction using a land use map, semi-supervised EM extraction and totally automatic EM extraction. These methods are described in the next sections (4.1, 4.2 and 4.3). The abundance of each EM (F_em_) has been retrieved by solving Eq. (11). Then, F_em_ values for green vegetation EMs have been taken as FVC values and compared against the *in situ* measurement. These results are reported in Section 4.4.

### 4.1. Endmember Extraction using a Land Use Map

The first EM extraction method considered in this paper is the easiest one, and it just consist on selecting on the image one pixel of bare soil and one pixel of green vegetation with a highest FVC (ideally with FVC = 100%).

**Figure 12.**
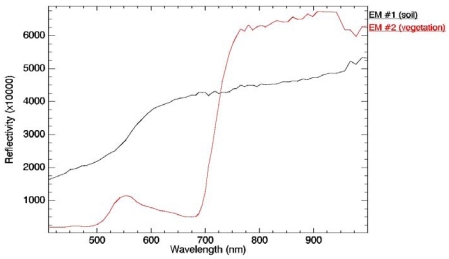
Reflectivity spectra for the endmembers extracted from the image using a land use map over a vegetation pixel and a bare soil pixel.

Despite that selection of these two pixels could be addressed using image-based data, for example taking into account some statistics for a VI such as the NDVI (in a similar way that the selection of NDVIs and NDVIv values discussed in Section 3.1), we have used the land use map of the test site and also the information provided by the FVC measured in situ (Table 1). Hence, a pixel of bare soil was selected in the surface between crops C1 and A1, whereas the green vegetation pixel was selected within the potatoes (P1) field (see map in Figure 4). Figure 12 shows the reflectivity spectra associated to these two pixels.

### 4.2. Endmember Extraction using the Pixel Purity Index (PPI)

One of the most successful semi-supervised algorithms for automatic endmember extraction in the literature has been the Pixel Purity Index (PPI) algorithm [4], which is quite popular in the remote sensing community due to its availability in the well-known Environment for Visualizing Images (ENVI) software package distributed by ITT Visual Information solutions (www.ittvis.com; formerly Research Systems, Inc. [23]). The algorithm proceeds by generating a large number of random, *N*-dimensional unit vectors called “skewers” so that every pixel (vector) in the hyperspectral scene is projected onto each skewer, and the data points that correspond to extrema in the direction of each skewer are identified and placed on a list. As more skewers are generated, the list grows, and the number of times a given pixel is placed on this list is also tallied. The pixels with the highest tallies are selected using a cut-off threshold parameter defined in advance by the user, and these pixels are then loaded into an *N*-dimensional visualization tool available in ENVI software [23]. This tool allows a trained analyst to select a final set of endmembers after an interactive process, in which selected pixels after applying the threshold above can be rotated and visualized in *N*-dimensional space, analyzing their convexity in the *N*-dimensional data cloud comprised by original pixel vectors.

In our experiments with the selected hyperspectral CHRIS data set, the PPI algorithm was run as follows. First, the virtual dimensionality (VD) concept [7] was used to estimate the number of endmembers in the data. According to the VD concept, which has been widely used to estimate the number of endmembers in hyperspectral scenes in previous work [21, 22], the number of endmembers in the data was 10. Then, we run the PPI with different number of skewers.

**Figure 13.**
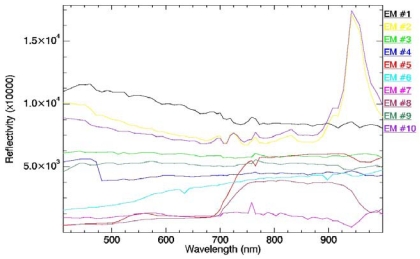
Reflectivity spectra for the endmembers extracted from the image using the PPI.

In our experiments, we observed that PPI produced essentially the same final set of endmembers for the considered scene when the number of skewers was above 3,000 (values of 1,000 and 10,000 were also tested). Based on the above simple experiment, the cut-off threshold parameter was set to the mean of PPI scores obtained after 3,000 iterations. These parameter values are in agreement with those used before in the literature [21, 22]. Pixels were then grouped into smaller subsets based on their clustering in the N‐dimensional space, using ENVI's N-dimensional visualization tool. Finally, resulting groups of extreme pixels were linked to the original image, and the mean spectrum of each group was used as a candidate endmember for spectral unmixing purposes. Figure 13 shows the reflectivity spectra for the 10 EMs extracted with the PPI procedure. In this case, EMs #5 and #8 correspond to green vegetation.

### 4.3. Automated Morphological Endmember Extraction (AMEE)

The reflectivity spectra for each endmember have been automatically extracted from the image using the AMEE (Automated Morphological Endmember Extraction) method. The input to AMEE method is the full spectral data cube, with no previous dimensionality reduction. The method is based on two parameters: a minimum S_min_ and a maximum S_max_ spatial kernel size. Firstly, a minimum kernel K=S_min_ is considered. This structuring element (SE) is moved through all the pixels of the image, defining a spatial context around each hyperspectral pixel. Let us denote by ***h***(x,y) the pixel vector at spatial coordinates. (x,y) The spectrally purest (***p***) and the spectrally most highly mixed (***m***) spectral signatures are respectively obtained at the neighborhood of ***h***(x,y) defined by K using two extended morphological operations [20]:

(13) $$\text{p}={\text{arg}\_\text{Max}}_{\left(\text{s},\text{t}\right)\in \text{K}}\;\left\{\sum_{\text{s}}\sum_{\text{t}}\text{dist}\left(\text{h}(\text{x},\text{y}),\text{h}(\text{x}-\text{s},\text{y}-\text{t})\right)\right\},\;\forall \left(\text{s},\text{t}\right)\in \text{K}$$

(14) $$\text{m}{=\text{arg}\_\text{Min}}_{\left(\text{s},\text{t}\right)\in \text{K}}\;\left\{\sum_{\text{s}}\sum_{\text{t}}\text{dist}\left(\text{h}(\text{x},\text{y}),\text{h}\left(\text{x}+\text{s},\text{y}+\text{t}\right)\right)\right\},\;\forall \left(\text{s},\text{t}\right)\in \text{K}$$

where *dist* is the spectral angle distance (SAD), a standard metric in hyperspectral analysis. A morphological eccentricity index (MEI) [21] is then obtained by calculating the SAD distance between the two above signatures. This operation is repeated for all the pixels in the scene, using SEs of progressively increased size, and the resulting scores are used to evaluate each pixel in both spatial and spectral terms. The algorithm performs as many iterations as needed until. K=S_max_ The associated MEI value of selected pixels at subsequent iterations is updated by means of newly obtained values, i.e. the values obtained for the same pixel at different iterations are accumulated (not replaced) in order to progressively consider a larger spatial context, until a final MEI image is generated. Endmember selection is performed by a fully automated approach consisting of two steps: 1) autonomous segmentation of the MEI image, and 2) spatial/spectral growing of resulting regions [20].

**Figure 14.**
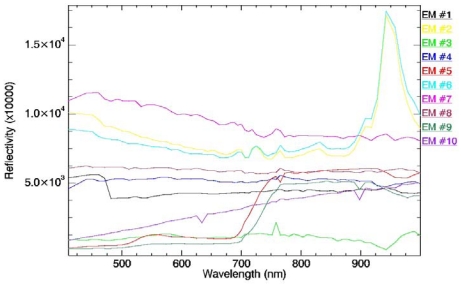
Reflectivity spectra for the endmembers extracted automatically from the image using the AMEE.

In this analysis, we have considered the following spatial kernels for the endmember search:, S_min_ a disk-shaped SE with radius of three pixels, and, S_max_ a disk-shaped SE with radius of 15 pixels. These parameter values have been reported in previous work [20] to provide satisfactory results in a wide range of applications. Using the parameter settings above, the AMEE method extracted a total amount of 10 EMs, in which 2 EMs for green vegetation have been found (the rest of the EMs correspond mainly to clouds, shadows and bare soil). Figure 14 illustrates the reflectivity spectra for the EMs extracted with the AMEE, in which EMs #5 and #9 correspond to green vegetation. EMs providing reflectivity values higher than 1 correspond to clouds, since they can not be atmospherically corrected when converting TOA reflectivities to at-surface ones.

### 4.4. Algorithms testing

**Figure 15.**
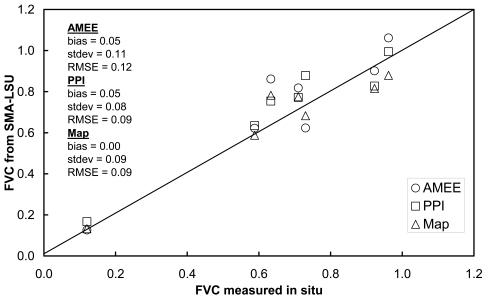
Comparison between the FVC retrieved from Spectral Mixture Analysis and Linear Spectral Unmixing (SMA-LSU) and the one measured in situ. In the SMA-LSU technique, endemembers have been extracted using the Automated Morphological Endmember Extraction (AMEE) method, the Pixel Purity Index (PPI) and the land use map of the study area (Map). ‘Bias’ is the mean difference between the retrieved FVC and the one measure in situ, ‘stdev’ is the standard deviation of the bias, and ‘RMSE’ is the Root Mean Square Error computed as square sum of the bias and the ‘stdev’.

FVC retrievals using abundance of green vegetation EMs extracted with the three different methods presented in the previous sections have been compared against the in situ measurements presented in Table 1. The results obtained in this test are presented in Figure 15. All the three methods considered for EM extraction provided RMSE < 12%, with the one based on the land use map providing the best results, with a zero bias and a RMSE < 9%, followed by the PPI method, also with a RMSE = 9% but a bias = 5%. The AMEE method provided a RMSE < 12%, again with a bias = 5%. The order for the three methods in terms of its accuracy is somehow expected, since the one providing the best results is totally supervised, i. e., it is not an automatic extraction since a land use map of the test site is required. It is followed by PPI, in which as semi-supervised procedure was considered as explained in Section 4.2. The last one is the AMEE method, which is totally automatic. Therefore, there is a compromise between accuracy and automatic (without dependence on external data) retrieval, like many times occur in algorithms applied to remote sensing data. Note that these methods, based on SMA-LSU techniques, are generally slighter accurate than the ones based on NDVI, GVI or GBVI. Only FVC retrievals based on the VARIgreen were more accurate, albeit slightly.

## 5. Summary and Conclusions

The fraction of vegetation cover or FVC is a key variable in many environmental studies. Different approaches have been published in order to retrieve this parameter from satellite data. Traditionally, these approaches have used relationships between FVC and vegetation indices. In this paper relationships between the FVC and the NDVI and VARIgreen indices (or its variants GVI and GBVI) adapted for CHRIS data have been analyzed and tested. Both provide good results, especially the FVC vs the VARIgreen approach, with RMSE values below 10%. NDVI based approaches provided a RMSE = 13%. The approach based on the VARIgreen has also the advantage of using TOA (Top Of Amosphere) data, so the atmospheric correction is not required. The availability of several spectral bands in the case of the CHRIS sensor, allows the application of other more sophisticated techniques for FVC retrieval, as for example Spectral Mixture Analysis or, more specifically and the one used in this paper, Linear Spectral Unmixing. This technique has been applied using three different methods for endemembers extraction: 1) an automatic procedure based on the AMEE method, 2) a semi-supervised procedure based on the PPI and 3) a totally supervised procedure using a land use map of the study area. Respectively, accuracies were 12%, 9% and 9%. It is important to remark that these results are only slightly better than the ones obtained from vegetation indices. Even FVC retrievals from VARIgreen provided better results, albeit also slightly.

Some issues are still open, and further research is required address them, as for example the application of the FVC retrieval methods presented in this paper to temporal series of remote sensing images in order to extract strong conclusions about the performance of each method, the angular sensitivity of both approaches based on vegetation indices and SMA-LSU, the comparison with other techniques for endmember extraction, or the influence of clouds in the image when extracting the endmembers.

## Figures and Tables

**Table 1.** Fractional vegetation cover measured in situ over different samples using hemispherical photographs (FVC_in situ_) and standard deviation values (σ).

**Sample**	**Notation**	**FVC_in situ_**	**σ**
Garlic	G1	0.12	0.09
Corn	C2	0.71	0.12
Corn	C1	0.63	0.08
Sugarbeet	B3	0.923	0.013
Alfalfa	A10	0.73	0.12
Alfalfa	A1	0.59	0.12
Potatoes	P1	0.96	0.04

**Table 2.** Mean NDVI and standard deviation (σ) values extracted from the CHRIS image acquired at near nadir view and using bands 48 (0.852 μm) and 25 (0.674 μm). Values were extracted for the whole plots associated to the different crops.

**Sample**	**Notation**	**NDVI**	**σ**
Garlic	G1	0.18	0.02
Corn	C2	0.792	0.014
Corn	C1	0.80	0.04
Sugarbeet	B3	0.791	0.013
Alfalfa	A10	0.72	0.05
Alfalfa	A1	0.67	0.06
Potatoes	P1	0.80	0.03

**Table 3.** Mean GVI, VARIgreen and GBVI values with their standard deviation (σ) extracted from the CHRIS image acquired at near nadir view and using bands 25 (0.674 μm, red), 14 (0.563 μm, green) and 8 (0.502 μm, blue). Values were extracted for the whole plots associated to the different crops. GVI and GBVI were obtained from at-surface reflectivities, whereas VARIgreen was obtained from TOA reflectivities.

**Sample**	**Notation**	**GVI**	**σ**	**VARIgreen**	**σ**	**GBVI**	**σ**
Garlic	G1	-0.156	0.011	-0.279	0.014	-0.221	0.015
Corn	C2	0.06	0.03	0.29	0.02	0.09	0.04
Corn	C1	0.01	0.03	0.28	0.07	0.01	0.03
Sugarbeet	B3	0.270	0.016	0.367	0.018	0.37	0.02
Alfalfa	A10	0.05	0.04	0.18	0.06	0.06	0.06
Alfalfa	A1	0.02	0.04	0.15	0.06	0.03	0.05
Potatoes	P1	0.30	0.05	0.45	0.06	0.39	0.06

**Table 4.** Statistics obtained in the test of the different approaches considered for retrieving Fractional Vegetation Cover (FVC) from Vegetation Indices (VI) using Eqs. (2), (8), (9) and (10). VIs and VIv refer respectively to ‘soil’ and ‘vegetation’ values associated with each VI. The assumption considered to extract VIs and VIv values is given in brackets. ‘Bias’ is the mean difference between retrieved FVC values and *in situ* ones, ‘stdev’ is the standard deviation of the bias, and RMSE is the Root Mean Square Error obtained as a square sum of ‘bias’ and ‘stdev’.

**VI**	**VIs**	**VIv**	**bias**	**stdev**	**RMSE**
NDVI	0.11 *(histogram)*	0.82 *(histogram)*	0.13	0.14	0.19
NDVI	-0.14 *(minimum)*	0.91 *(maximum)*	0.11	0.12	0.17
NDVI	0.11 *(histogram)*	0.91 *(maximum)*	0.04	0.12	0.13
NDVI	0.15 *(global)*	0.90 *(global)*	0.04	0.13	0.13
NDVI	0.08 *(in situ)*	0.98 *(in situ)*	0.00	0.13	0.13

GVI	-0.34 *(minimum)*	0.41 *(maximum)*	-0.11	0.11	0.16
GVI	-0.16 *(histogram)*	0.41 *(maximum)*	-0.25	0.10	0.27
GVI	-0.33 *(in situ)*	0.28 *(in situ)*	0.00	0.11	0.11

VARIgreen	-0.36 *(minimum)*	0.54 *(maximum)*	-0.04	0.07	0.08
VARIgreen	-0.31 *(histogram)*	0.54 *(maximum)*	-0.06	0.07	0.10
VARIgreen	-0.38 *(in situ)*	0.50 *(in situ)*	0.00	0.08	0.08

GBVI	-0.45 *(minimum)*	0.49 *(maximum)*	-0.08	0.10	0.13
GBVI	-0.24 *(histogram)*	0.49 *(maximum)*	-0.20	0.10	0.22
GBVI	-0.44 *(in situ)*	0.38 *(in situ)*	0.00	0.11	0.11

**Table 5.** Percentage (in %) of Fractional Vegetation Cover (FVC) angular variations (value at certain view angle minus value at nadir divided by the value at nadir) for the different plots. Mean and standard deviation (std dev) values are also given. PROBA/CHRIS view angles are expressed as Fly-by Zenith Angles (FZA) and also as View Zenith Angles (VZA).

**FVC from NDVI**								

**FZA (°)**	**VZA (°)**	**G1**	**C2**	**C1**	**B3**	**A10**	**A1**	**P1**
-55	-57.4	26.5	-1.6	-10.2	-1.6	-2.7	0.1	-4.5
-36	-42.5	4.3	3.1	-8.3	2.3	0.8	-1.3	-1.9
0	27.6	0.0	0.0	0.0	0.0	0.0	0.0	0.0
36	42.4	-1.5	1.4	3.7	0.8	4.0	2.6	-2.4
55	57.3	37.7	2.4	1.0	-1.9	-0.1	5.3	-4.8
	**mean**	**13.4**	**1.1**	**-2.8**	**-0.1**	**0.4**	**1.3**	**-2.7**
	**std dev**	**17.6**	**1.9**	**6.1**	**1.7**	**2.4**	**2.6**	**2.0**
**FVC from VARIgreen**								

**FZA (°)**	**VZA (°)**	**G1**	**C2**	**C1**	**B3**	**A10**	**A1**	**P1**

-55	-57.40	26.5	-0.9	-15.7	-6.0	-2.1	4.5	-10.4
-36	-42.53	10.1	9.5	-12.4	4.0	4.8	2.1	-2.4
0	27.60	0.0	0.0	0.0	0.0	0.0	0.0	0.0
36	42.44	1.7	-1.5	2.2	-3.3	3.1	0.8	-7.9
55	57.29	24.5	-1.1	-2.9	-9.9	-2.5	7.6	-13.5
	**mean**	**12.6**	**1.2**	**-5.8**	**-3.0**	**0.7**	**3.0**	**-6.8**
	**std dev**	**12.4**	**4.7**	**7.9**	**5.3**	**3.2**	**3.1**	**5.6**
